# Supplementary material for: The effect of multiple interventions to balance healthcare demand for controlling COVID-19 outbreaks: a modelling study
Source: Sci Rep. 2021 Feb 4;11:3110. doi: 10.1038/s41598-021-82170-y (PMC7862317; doi:10.1038/s41598-021-82170-y)
Supplement: Supplementary file 1 — Supplementary information. [file 41598_2021_82170_MOESM1_ESM.docx]

# Supplementary Materials

**Transmission model structure**

We estimated changes in COVID-19 transmissibility over time via the effective reproduction number ($R_{t}$), which represents the mean number of secondary infections that result from a primary case of infection at time t. Values of $R_{t}$ exceeding 1 indicate that the epidemic will tend to grow, whereas values below 1 indicate that the epidemic will tend to decline. We estimated the time-varying reproduction numbers from serial intervals and incidence of COVID-19 cases over time. The transmission rate β(t) is related to the basic reproduction number by the formula

$$\beta\left( t \right)= \gamma R_{t}$$

We used particle filter simulation to fit the real (Rt),the specific steps are:

First step:

1. Generate random initial particles according to the initial state-particle initialization(Our initial R0=3),generally generated using Gaussian random distribution
2. Calculate the initial measurement value from the initial state according to the measurement function equation

Second step(Enter the iteration period):

1. Calculate the current state from the initial state according to the state transition matrix or equation
2. Calculate the current observation value from the current state according to the measurement state matrix or equation
3. Generate particles based on the number of particles(We set NN=1000):
4. Generate particles based on the number of particles
5. Calculate the current observation value of the particle according to the measurement function matrix or equation
6. Calculate the likelihood function value to get the particle weight value
7. Normalize the weights of all particles(so that the weight of all particles are between 0-1)
8. Random importance resamples all particles to obtain subscripts with random numbers greater than the limit of particle weights
9. Reassign the current particle sample to the new particle based on the subscript
10. The state is estimated as the mean of the new particles

We implemented a modified SEIR model to account for a dynamic Susceptible [S], Exposed [E] (infected but asymptomatic), Infectious [I] (infected and symptomatic) and Recovered [R] or Deceased [D] population’s state. In order to estimate health demand, we categorized the infectious group into two sub-cases: Mild [M] and Critical [C]; where Mild cases do not need hospital beds; Critical cases need hospital beds but possibly cannot get it due to shortage of health sources.

Following previous assumptions, the implementation of dynamic transmission of our modified SEIR model follows steps as below:

$$S_{t+1}=S_{t}-\frac{\beta_{1}M_{t}I_{t}S_{t}}{N_{t}}-\frac{{i\beta}_{2}M_{t}I_{t}S_{t}}{N_{t}}$$

$$E_{t+1}=E_{t}+\frac{\beta_{1}M_{t}I_{t}S_{t}}{N_{t}}+\frac{{i\beta}_{2}M_{t}I_{t}S_{t}}{N_{t}}-\varphi_{1}E_{t}-\gamma_{1}E_{t}$$

$$M_{t+1}=M_{t}+\varphi_{1}E_{t}-\varphi_{2}{(\frac{s+c}{m})M}_{t}-\gamma_{2}M_{t}$$

$$\mathrm{If}C_{t}>B_{t}J_{t}H_{t} :$$

$${NH}_{t}={C_{t}-B}_{t}J_{t}H_{t}$$

$${IH}_{t}=B_{t}J_{t}H_{t}$$

$$\mathrm{else}$$

$${NH}_{t}=0$$

$${IH}_{t}= C_{t}$$

$$C_{t+1}=C_{t}+\varphi_{2}{\left( \frac{s+c}{m} \right)M}_{t}-\gamma_{3}\mathrm{NH}_{t}-\gamma_{4}O\mathrm{IH}_{t}-\gamma_{5}(1-O)\mathrm{IH}_{t}-\delta_{1}\left( \frac{c}{s+c} \right)\mathrm{NH}_{t}-\delta_{2}\left( \frac{c}{s+c} \right)O\mathrm{IH}_{t}-\delta_{3}\left( \frac{c}{s+c} \right)(1-O)\mathrm{IH}_{t}$$

$$I_{t+1}=M_{t+1}+C_{t+1}$$

$$D_{t+1}=D_{t}+\delta_{1}\left( \frac{c}{s+c} \right)\mathrm{NH}_{t}+\delta_{2}\left( \frac{c}{s+c} \right)O\mathrm{IH}_{t}+\delta_{3}\left( \frac{c}{s+c} \right)(1-O)\mathrm{IH}_{t}$$

$$R_{t+1}=R_{t}+\gamma_{1}E_{t}+\gamma_{2}M_{t}+\gamma_{3}\mathrm{NH}_{t}+\gamma_{4}\mathrm{OIH}_{t}+\gamma_{5}{(1-O)\mathrm{IH}}_{t}$$

Here S(t) is the number of individuals in UK susceptible at time t, E(t) is the number of people in the UK who have been infected but asymptomatic, I(t) is the number of people in the UK who have been infected and symptomatic, M(t) is the number of people with mild disease, C(t) is the number of people with severe and critical disease, and NH(t) is the number of people with severe and critical disease who have not been hospitalized, IN(t) is the number of people with severe and critical disease who have been hospitalized, R(t) is the number of patients who have been cured, D(t) is the number of patients who have died.

Parameter *i* is the efficiency of isolation contacts. Parameter m is the proportion of mild case, parameter s is the proportion of severe case, and parameter c is the proportion of critical case. Parameter O is the percentage of people over 65 in the UK.

Parameter $\beta_{1}$ is the transmission rate from I to S, Parameter $\beta_{2}$ is the transmission rate from E to S. Parameter $\varphi_{1}$ is the transmission rate from E to M (1/$\alpha_{1}$(incubation period)), Parameter $\varphi_{2}$ is the transmission rate from M to C (1/$\alpha_{2}$(average period from M to C)).

Parameter $\gamma_{1}$ is the transmission rate from E to R (1/$ɤ_{1}$(average period from E to R)), parameter $\gamma_{2}$ is the transmission rate from M to R (1/$ɤ_{2}$(average period from M to R)), parameter $\gamma_{3}$ is the transmission rate from NH to R (1/$ɤ_{3}$(average period from NH to R)), parameter $\gamma_{4}$ is the transmission rate of older people from IH to R (1/$ɤ_{4}$(average period of older people from IH to R)), parameter $\gamma_{5}$ is the transmission rate of non-older people from IH to R (1/$ɤ_{5}$(average period of non-older people from IH to R)).

Parameter $\delta_{1}$ is the transmission rate from NH to R (1/$d_{1}$(average period from NH to D)), parameter $\delta_{2}$ is the transmission rate of older people from IH to R (1/$d_{2}$(average period of older people from IH to D)), parameter $\delta_{3}$ is the transmission rate of non-older people from IH to R (1/$d_{3}$(average period of non-older people from IH to D)).

Parameter $B_{t}$ is the number of hospital beds in the UK, parameter $J_{t}$ is the percentage of available hospital beds for COVID-19 critical cases, $H_{t}$ is the percentage of unoccupied hospital beds，$M_{t}$ is the intensity of intervention.

**Data and code availability**

All data and code required to reproduce the analysis is available at:

<https://github.com/TurtleZZH/Comparison-of-Multiple-Interventions-for-Controlling-COVID-19-Outbreaks-in-London-and-the-UK>

**Table of data on multiple interventions in the UK**

Days: Number of days from February 6^th^. Q: The number of total infected population. Z: The number of accumulative infected population. E: The number of daily exposed population. RI: The number of real infected population. D: The number of total deceased population. Rate: Mortality rate. PTnH: Peak time of non-hospital population. PnH: Peak value of non-hospital population. Rt: Basic reproduction number)

| **Interventions** | **Days** | **Date** | **Q** | **Z** | **E** | **RI** | **D** | **Rate** | **PTnH** | **PnH** | **Rt** |
| --- | --- | --- | --- | --- | --- | --- | --- | --- | --- | --- | --- |
| All UK Suppression  (Outbreak period 28-51) | 28 | 03/05 | 2,202 | 1,484 | 3,060 | 51 | 3 |  | 57 | 13,920 | 2.31 |
|  | 46 | 03/23 | 240,768 | 151,038 | 157,950 | 6,650 | 536 |  |  |  | 1.08 |
|  | 51 | 03/28 | 525,723 | 319,257 | 245,911 | 17,089 | 1,521 |  |  |  | 0.73 |
|  | 200 | 08/24 | 4,705,965 | 2,152,160 | 18,015 |  | 69,511 |  |  |  | 1.01 |
|  | 250 | 10/13 | 4,949,123 | 2,255,361 | 7,901 |  | 74,087 |  |  |  |  |
|  | 350 |  | 5,101,783 | 2,320,087 | 1,496 |  | 76,972 | 1.5% |  |  |  |
| All UK Mitigation (10)  (Outbreak period 50-80) | 46 | 03/23 | 240,768 | 151,038 | 157,950 | 6,650 | 536 |  | 93 | 1,004,000 | 1.47 |
|  | 50 | 03/27 | 458,202 | 280,725 | 267,428 | 14,543 | 1,257 |  |  |  | 1.44 |
|  | 80 | 04/27 | 16,840,236 | 93,406,165 | 384,2315 |  | 369,577 |  |  |  | 0.88 |
|  | 130 | 06/15 | 45,366,239 | 20,790,323 | 110,916 |  | 2,738,416 |  |  |  | 0.94 |
|  | 150 | 07/05 | 46,084,841 | 20,992,335 | 26,941 |  | 2,831,113 |  |  |  | 1.36 |
|  | 350 |  | 46,345,452 | 21,066,117 | 0 |  | 2,844,119 | 6.13% |  |  |  |
| All UK Mitigation (8) (Outbreak period 42-92) | 42 | 03/19 | 113,673 | 74,069 | 94,475 | 144 | 190 |  | 106 | 589,000 | 1.42 |
|  | 46 | 03/23 | 24,0768 | 151,038 | 15,7950 | 6,650 | 536 |  |  |  | 1.24 |
|  | 92 | 05/09 | 14,787,392 | 7,713,881 | 1,974,356 |  | 430,664 |  |  |  | 0.95 |
|  | 150 | 07/05 | 36,316,946 | 16,671,750 | 154,986 |  | 2,075,255 |  |  |  | 0.89 |
|  | 180 | 08/04 | 37,469,894 | 17,069,757 | 32,987 |  | 2,158,750 |  |  |  | 1.06 |
|  | 350 |  | 37,813,104 | 17,187,789 | 10 |  | 2,170,559 | 5.74% |  |  |  |
| All UK Mitigation (6) (Outbreak period 36-110) | 36 | 03/13 | 23,598 | 15,890 | 33,006 | 798 | 32 |  | 125 | 234,500 | 1.48 |
|  | 46 | 03/23 | 240,768 | 151,038 | 157,950 | 6,650 | 536 |  |  |  | 1.10 |
|  | 110 | 05/27 | 10,365,106 | 5,100,766 | 695,450 |  | 331,631 |  |  |  | 0.98 |
|  | 250 | 10/13 | 25,022,270 | 11,395,381 | 25,679 |  | 1,150,475 |  |  |  |  |
|  | 300 | 12/02 | 25,313,038 | 11,511,169 | 6,169 |  | 1,157,223 |  |  |  |  |
|  | 350 |  | 25,382,757 | 11,538,870 | 1,469 |  | 1,158,855 | 4.56% |  |  |  |
| London Suppression  and Non-London Rolling (Outbreak period 28-51) | 28 | 03/05 | 2,202 | 1,484 | 3,060 | 51 | 3 |  | 57 | 13,920 | 2.38 |
|  | 46 | 03/23 | 240,768 | 151,038 | 157,950 | 6,650 | 536 |  |  |  | 1.08 |
|  | 51 | 03/28 | 525,723 | 319,257 | 239,169 | 245,911 | 1,521 |  |  |  | 0.68 |
|  | 200 | 08/24 | 7,538,809 | 3,468,537 |  | 65,888 | 111,619 |  |  |  | 1.03 |
|  | 250 | 10/13 | 8,535,877 | 3905,877 |  | 41,630 | 128,552 |  |  |  |  |
|  | 350 |  | 9,367,882 | 4,265,557 |  | 9,485 | 143,105 | 1.52% |  |  |  |
| London and  Non-London Rolling  (Outbreak period 28-51) | 28 | 03/05 | 2,202 | 1,484 | 3,060 | 51 | 3 |  | 57 | 13,920 | 2.31 |
|  | 46 | 03/23 | 240,768 | 151,038 | 157,950 | 6,650 | 536 |  |  |  | 1.08 |
|  | 51 | 03/28 | 525,723 | 319,257 | 25,911 | 17,089 | 1,521 |  |  |  | 0.69 |
|  | 250 | 10/13 | 914,6763 | 4,185,793 | 4,232 |  | 138,853 |  |  |  |  |
|  | 280 | 11/12 | 955,0111 | 4,358,435 | 23,713 |  | 145,921 |  |  |  |  |
|  | 350 |  | 1,0042,694 | 4,572,780 | \| 10,079 \| \| --- \| |  | 154,569 | 1.53% |  |  |  |

**Table 1.** Data on different measures in the UK. (Days: Days from February 6^th^.Q: The number of total infected population. Z: The number of accumulative infected population. E: The number of daily exposed population. RI: The number of real infected population. D: The number of total deceased population. Rate: Mortality rate. PTnH: Peak time of non-hospital population. PnH: Peak value of non-hospital population. Rt: Basic reproduction number)

| Multiple interventions | U/M | E | TI | TD | PTSC | PHSC | PHnT | PnH | FMR |
| --- | --- | --- | --- | --- | --- | --- | --- | --- | --- |
| All UK suppression | U | Y | 5,101,783 | 76,972 | 70 | 61,360 | 57 | 13,920 | 1. 5% |
| All UK 3 weeks rolling (3 and 4) | U | Y | 6,896,541 | 102,871 | 70 | 61,360 | 57 | 13,920 | 1.49% |
| All UK 2 weeks rolling (3 and 5) | U | Y | 10,116,715 | 160,236 | 84 | 73,660 | 57 | 13,920 | 1.58% |
| All UK 3 weeks rolling (3 and 5) | U | Y | 10,042,694 | 154,569 | 97 | 73,560 | 57 | 13,920 | 1.53% |
| All UK 4 weeks rolling (3 and 5) | U | Y | 9,925,852 | 151,164 | 111 | 72,420 | 57 | 13,920 | 1.52% |
| All UK 2 weeks rolling (3 and 6) | M | N | 14,159,946 | 325,904 | 112 | 95,650 | 112 | 28,990 | 2.30% |
| All UK 3 weeks rolling (3 and 6) | M | N | 14,228,064 | 319,955 | 139 | 97,110 | 139 | 30,450 | 2.24% |
| All UK 4 weeks rolling (3 and 6) | M | N | 14,228,569 | 310,589 | 113 | 101,700 | 113 | 35,090 | 2.18% |
| All UK 3 weeks rolling (3 and 8) | M | N | 23,351,902 | 971,622 | 139 | 2,287,000 | 139 | 162,000 | 4.16% |
| London suppression (3), other regions 2 weeks rolling (3 and 5) | U | Y | 9,427,917 | 147,394 | 84 | 70,410 | 57 | 13,920 | 1.56% |
| London suppression (3), other regions 3 weeks rolling (3 and 5) | U | Y | 9,367,882 | 143,105 | 96 | 68,940 | 57 | 13,920 | 1.52% |
| London suppression (3), other regions 4 weeks rolling (3 and 5) | U | Y | 9,268,946 | 140,900 | 110 | 66,660 | 57 | 13,920 | 1.52% |
| London All suppression -- nonLondon 3-5 3 weeks rolling | U | Y | 8,441,584 | 124,757 | 70 | 61,360 | 57 | 13,920 | 1.47% |
| Six weeks suppression,then high intensity mitigation 3-5 | M | N | 1,562,2092 | 302,186 | 184 | 82,650 | 187 | 16,380 | 1.93% |
| Six weeks suppression,then high intensity mitigation 3-6 | M | N | 23,685,805 | 878,121 | 180 | 173,500 | 180 | 106,800 | 3.70% |
| Six weeks suppression,then moderate intensity mitigation 3-7 | M | N | 30,647,196 | 1,451,931 | 167 | 314,100 | 167 | 247,500 | 4.73% |
| Six weeks suppression,then moderate intensity mitigation 3-8 | M | N | 36,440,443 | 1,929,340 | 155 | 488,600 | 155 | 422,000 | 5.29% |
| Six weeks suppression,then 3 week rolling 3-5 | U | Y | 8,951,775 | 132,121 | 70 | 61,360 | 57 | 13,920 | 1.47% |
| Six weeks suppression,then 3 week rolling 3-6 | M | N | 13,092,598 | 210,152 | 161 | 77,220 | 57 | 13,920 | 1.60% |
| Six weeks suppression,then 3 week rolling 3-7 | M | N | 17,696,613 | 467,888 | 161 | 121,600 | 161 | 54,950 | 2.64% |
| Six weeks suppression,then 3 week rolling 3-8 | M | N | 22,339,206 | 816,746 | 161 | 129,800 | 161 | 126,100 | 3.65% |
| Six weeks suppression,then 2 week rolling 3-5 | M | N | 8,694,038 | 128,310 | 70 | 61,360 | 57 | 13,920 | 1.47% |
| Six weeks suppression,then 2 week rolling 3-6 | M | N | 12,632,168 | 187,928 | 167 | 68,290 | 57 | 13,920 | 1.48% |
| Six weeks suppression,then 2 week rolling 3-7 | M | N | 17,078,493 | 409,394 | 168 | 104,500 | 168 | 37,860 | 2.39% |
| Six weeks suppression,then 2 week rolling 3-8 | M | N | 21,555,482 | 736,153 | 168 | 160,100 | 168 | 93,430 | 3.41% |
| Six weeks suppression,then 4 week rolling 3-5 | U | Y | 9,201,486 | 135,590 | 70 | 61,360 | 57 | 13,920 | 1.47% |
| Six weeks suppression,then 4 week rolling 3-6 | M | N | 13,468,065 | 238,766 | 128 | 84,680 | 128 | 18,020 | 1.77% |
| Six weeks suppression,then 4 week rolling 3-7 | M | N | 18,204,675 | 521,281 | 181 | 132,000 | 181 | 65,330 | 2.86% |
| Six weeks suppression,then 4 week rolling 3-8 | M | N | 22,937,138 | 882,755 | 181 | 1,960,000 | 181 | 129,400 | 3.84% |
| Nine weeks suppression,then 2 week rolling 3-5 | U | Y | 7,761,765 | 114,503 | 70 | 61,360 | 57 | 13,920 | 1.47% |
| Nine weeks suppression,then 3 week rolling 3-5 | U | Y | 7,980,859 | 117,608 | 70 | 61,360 | 57 | 13,920 | 1.47% |
| Nine weeks suppression,then 4 week rolling 3-5 | U | Y | 8,166,998 | 120,494 | 70 | 61,360 | 57 | 13,920 | 1.47% |
| Eight weeks suppression,then 2 week rolling 3-5 | U | Y | 8,057,850 | 118,845 | 70 | 61,360 | 57 | 13,920 | 1.47% |
| Eight weeks suppression,then 3 week rolling 3-5 | U | Y | 8,289,685 | 122,218 | 70 | 61,360 | 57 | 13,920 | 1.47% |
| Eight weeks suppression,then 4 week rolling 3-5 | U | Y | 8,494,280 | 125,231 | 70 | 61,360 | 57 | 13,920 | 1.47% |

Table 2. Data on different suppression intervention times in the UK. (FMR: Final morality rate = Total deaths / Total infections. PHSC: Peak value of healthcare demand (Severe and Critical cases), PTSC: Peak time of healthcare demand; PnH: Peak value of non-hospital population, PTnH: Peak time of non-hospital population; TD: Total deaths (UK), TI = Total infections (UK), E: End in 1 year, D: Distribution (Unimodal/Multimodal))
